# Supplementary material for: Pneumococcal Pneumonia and Invasive Pneumococcal Disease in Those 65 and Older: Rates of Detection, Risk Factors, Vaccine Effectiveness, Hospitalisation and Mortality
Source: Geriatrics (Basel). 2021 Feb 4;6(1):13. doi: 10.3390/geriatrics6010013 (PMC7931064; doi:10.3390/geriatrics6010013)
Supplement: Supplementary file 1 [file geriatrics-06-00013-s001.pdf]

Table 1. Data sources

Author and date	Data source
Section 3.1 Literature review	
Remschmidt 2016 [8]	Systematic review
Falkenhorst 2017 [9]	Systematic review
Berold 2018 [10]	Systematic review
Section 3.2 Prevalence of pneumococcal disease	
US Centers for Medicare and Medicaid Services 2020 [13]	National database
Chalmers 2016 [14]	Systematic review
Van Hoek 2012 [15]	National data bases (Health and Protection Agency, UK)
Waight 2015 [16]	Observational cohort study
LeBlanc 2019 [17]	National database (Canadian Immunization Research network)
Desmet 2021 [18]	National database (Belgian laboratory-based surveillance of invasive pneumococcal disease)
Section 3.3 Pneumococcal vaccination rates	
US Centers for Disease Control 2020 [19]	National database (Vaccination Coverage Among Adults Aged 65 and Over: United States, 2015)
Shen 2019 [20]	National database (Vaccination Medicare fee-for-service beneficiaries)
Black 2017 [21]	National databases (Centers for Medicare and Medicaid Services: CMS) Minimum Data Set (MDS). Influenza and pneumococcal vaccination status assessed for all residents of CMS-certified nursing homes using data reported to the MDS by all certified facilities
Vila-Corcoles 2019 [22]	Database, Autonomous Region of Catalonia, Spain
Wang 2020 [23]	Retrospective cohort study of 3 databases (Shanghai Chronic Disease Management Information System, Shanghai Immunization Program Information System, Hospital Record System at Shanghai Health Commission for Hospital Records)
Djennad 2019 [24]	National database (Public Health England, national surveillance of IPD)
Naito 2020 [25]	National databases (5-year Japanese national PPV23 immunization program; number of vaccines shipped to each municipality by pharmaceutical company MSD K.K. (Tokyo); Annual number of individuals ≥ 65 years, Statistics Bureau of the Ministry of Internal Affairs and Communications)

Murakami 2019 [26]	Postal and web-based nationwide survey to all municipalities in Japan, June 2016 (n=1741)
Public Health England 2018 [27]	National database (Pneumococcal polysaccharide vaccine (ppv) coverage report, England, April 2017 to March 2018)
Dyda 2016 [28]	Systematic review
Yang 2016 [29]	Analysis of introduction of PPV23 for seniors into Korean national immunization program May 2013

Section 3.4 Detection of pneumococcal pathogens in adults hospitalised for CAP

Isturiz 2019 [30]	Prospective cohort study (212 hospitals in 10 US states)
Daniel 2018 [31]	Prospective cohort study (2 hospitals in Nottingham, UK)
Htar 2017 [32]	Systematic review

3.5. Effect of vaccination on antibiotic resistance to medications used for pneumococcal disease

Htar 2017 [33]	Systematic review

3.6. Effect of vaccination on IPD caused by antibiotic-resistant pneumococcus

Htar 2019 [34]	Systematic review
Vadlamudi 2019 [35]	Systematic review
Vestjens [36]	National database (Netherlands Reference Laboratory)

3.7. The effect of pneumococcal vaccination on carriage rates in those ≥ 65

Van Deursen 2018 [37]	RCT (CAPiTA trial , Netherlands)
Azarian 2018 [38]	3 prospective observational cohort studies

3.8. Persistence of immunity following conjugate and polysaccharide vaccination in seniors

MacIntyre 2019 [39]	RCT
Musher 2010 [40]	RCT
Clutterbuck 2012 [41], Lazarus 2011 [42]	RCT
Lode 2011 [43]	RCT
Namkoong 2015 [44]	RCT

3.9. Effect of pneumococcal vaccination on the incidence of pneumococcal pneumonia, invasive pneumococcal disease, hospitalization and death

Hechter 2012 [45]	Prospective cohort study
Webber 2014 [46]	RCT
Ochoa-Gondar 2014 [47]	Prospective cohort study
Maruyama 2010 [48]	RCT

3.10. Studies which performed analyses controlling for risk factors

Tsai 2015 [51]	National databases (Taiwan National Health Insurance Research Database; Taiwan Ministry of Health Cause-of-Death Registration Database,

	and Invasive Pneumococcal Disease (IPD) Notification Database)
Suaya 2018 [52]	RCT (CAPiTA trial, Netherlands)

3.11 Studies which performed stratified analyses by age groups 65-74, 75-84, 85-89, and 90+

Van Werkhoven 2013 [53]	RCT (CAPiTA trial, Netherlands)
